# Editorial: Domestic violence and abuse: increasing global and intersectional understanding

**DOI:** 10.3389/frhs.2024.1465688

**Published:** 2024-09-12

**Authors:** Michelle Myall, Sara Morgan, Steph Scott

**Affiliations:** ^1^ School of Health Sciences, University of Southampton, Southampton, United Kingdom; ^2^ NIHR Applied Research Collaboration Wessex, Southampton, United Kingdom; ^3^ Centre for Population Health Sciences, University of Southampton, Southampton, United Kingdom; ^4^ Population Health Sciences Institute, Newcastle University, Newcastle-Upon-Tyne, United Kingdom

**Keywords:** domestic violence and abuse, intimate partner violence, family violence, intersectionality, under-represented communities, seldom heard groups

Editorial on the Research Topic Domestic violence and abuse: increasing global and intersectional understanding

## Introduction

Domestic violence and abuse (DVA) is a significant public health and social care challenge worldwide, with a myriad of associated physical, mental health and social care consequences. Globally, it is estimated that one in three women and one in six men will experience DVA in their lifetime. Further, lifetime prevalence is anticipated to be particularly high in less developed countries and particularly problematic for under-represented groups or those already experiencing health inequalities.

DVA intersects cultural, religious, sex, gender, age and ethnic boundaries and can include physical, sexual, economic, psychological, emotional violence and/or abuse, as well as controlling, coercive, violent or threatening behaviour, between people who are, or who have been, intimate partners or family members.

The five papers in our Research Topic highlight the diversity and breadth of work undertaken in DVA and explore DVA experiences across and between multiple intersections that advance understanding of it as a global and intersectional issue.

## Summary of papers

Fisher et al. evaluate a whole-of-hospital response at Royal Melbourne Hospital to family violence- a national concern that disproportionately affects those from marginalised communities. Between baseline (*n* = 526) and follow-up (*n* = 534) of an online survey they found an increase in screening of family violence alongside improved confidence and knowledge of family violence. Despite indications of positive change, the COVID-19 pandemic led to fewer staff trained and staff redeployments. This may have resulted in the low response rates observed among nursing (12%) and medical staff (10%) at follow-up. These rates meant that the full impact of the response could not be interpreted. The authors point to further research that may shed light on the extent of the hospital “transformation” including, for example, the use of family violence alerts on patient files by clinicians. Data linkages with the police and community service partners are recommended and would further support a demonstration of impact for patients affected by family violence.

Toccalino et al. review the triple intersection between brain injury (BI), mental health (MH) and intimate partner violence (IPV). The authors explore this largely overlooked academic literature – mostly published in the USA. Although they do not set out to identify explanations and/or causal mechanisms for these relationships, their results highlight areas that require further investigation including, how BI is identified among survivors of IPV, how survivors experience healthcare and intersectionality. The authors acknowledge their underlying assumption, that self-reported strangulation leads to BI, but do not state whether the samples are representative of all IPV survivors, even in the USA. This could inform a wider national debate around sexual education, and how to minimise exposure to pornography; known to promote harmful sexual practices, such as strangulation.

Children and young people who grow up in families where they are exposed to DVA are more at risk of perpetrating or experiencing violence later in life. Harris et al. identified, critically appraised and compared ten existing measures of childhood exposure to DVA, only four of which have been validated cross-culturally - The Child Exposure to Domestic Violence Scale, Children's Perception of Interparental Conflict Scale, Juvenile Victimization Questionnaire and The Violence Exposure Scale for Children. Crucially, the authors identify that there is no standardised measure for childhood exposure to DVA and that this is exacerbated in LMICs, leading to gaps in data and practice, and further widening of health inequalities.

Meanwhile, the brief research report by Rutter explores how mothers made meaning of their lives and identity when their parenting journey was disrupted by violence and abuse perpetrated by their adolescent children. Help-seeking behaviours were often met by denial, avoidance or blame from professionals unless they were familiar with the mother through her professional role, for example as a social worker or healthcare practitioner. This parent blaming serves to perpetuate victim/mother blaming narratives and holds mothers accountable for their children's abusive and violent behaviours rather than shifting the focus to how health, social care, police and education services could improve their response. Rutter calls for practitioners to be aware of their biases when working with families and for services to work together better. The report highlights a need for further research exploring how more consistent multi-agency support pathways can enable mothers to navigate structural challenges experienced when seeking support for adolescent-to-parent violence and abuse.

In their paper examining the prevalence of intimate partner violence (IPV) between bisexual men and between gay men living in the United States, Rustagi et al. continue the theme of marginalisation and stigma. Using an online survey to measure nine items from the Everyday Discrimination Scale, and applying the Minority Stress Model, they observed the cross-sectional association between perceived discrimination and three forms of IPV: sexual, physical and non-physical. Results showed a statistically significant association between these and suggested a higher IPV prevalence amongst bisexual men compared to gay men. Relationship status was also a risk factor and the need to reduce minority stress in sexual minority men was highlighted as a priority. The need for qualitative research to capture victim-survivor narratives on their experiences is also highlighted. The authors call for healthcare providers to tailor specific services for gay and bisexual men and the need for increased awareness raising and training to be able to screen and respond to them.

## Conclusion

While the papers in the Research Topic address a variety of issues, there are common threads between them. These highlight the global disproportional impact of DVA on those populations already facing disadvantage and discrimination, reveal the hidden victims of DVA and those who are less visible in mainstream discourse, policy and practice. This includes children, who are now recognised as victims in their own right. The papers point to DVA being preventable and key to this is the provision of specific, tailored services and early intervention delivered through a more joined-up, cross-sector and multi-agency response.

